# Neuropsychological impairment in bipolar affective disorder

**DOI:** 10.4103/0019-5545.46075

**Published:** 2005

**Authors:** Mubeen Taj, R. Padmavati

**Affiliations:** * Consultant Psychiatrist, Schizophrenia Research Foundation, Chennai, India; ** Deputy Director, Schizophrenia Research Foundation, Chennai, India

**Keywords:** Cognitive dysfunction, bipolar disorder, remission, attention, memory, executive function

## Abstract

**Background:**

Recent neurocognitive investigations during the euthymic phase of bipolar affective disorder have shown persistent cognitive deficits in 32% of patients. There is limited evidence in the Indian literature in this area.

**Aim:**

To study the neuropsychological functions in patients with bipolar affective disorder in remission.

**Methods:**

Thirty patients with bipolar disorder in remission were compared with 30 normal subjects on tests of attention, learning, memory and executive functions. Neuropsychological measures of the two groups were compared using the chi-square and Student t tests. This study was conducted in the outpatient department of the Schizophrenia Research Foundation.

**Result:**

Patients with bipolar disorder, in remission, have neuropsychological impairment in attention, memory and executive functioning.

**Conclusion:**

Cognitive dysfunction in patients with bipolar disorder in remission can contribute to social and occupational difficulties, reduced insight, increased risk of non-adherence and relapse.

## INTRODUCTION

Investigations of patients with bipolar affective disorder have demonstrated that although more than 97% of patients appear to recover clinically within 2 years, only 37% recover functionally during the same period.^1^ Vieta and MartinezAran suggest that residual cognitive dysfunction may contribute to this.^2^ Several studies using standardized diagnostic criteria and research designs have demonstrated neuropsychological impairment in patients with bipolar disorder in the euthymic phase.^3^ The presence of subclinical psychopathology in the euthymic phase could partially account for the observed residual deficits.

Frequently studied neuropsychological functions are attention, learning, working memory and executive function. Patients with bipolar disorder performed worse than normal controls on tests of executive function in a study which controlled for age, depressive symptoms and premorbid intelligence.^4^ Tham *et al*^5^ demonstrated a lowered performance in synonym reasoning. van Gorp *et al*.^6^ demonstrated that patients with euthymic bipolar disorder performed worse than normal controls in tests of declarative memory. Zubieta *et al*.^7^ showed poorer performance on measures of verbal learning, executive function and motor coordination in patients with bipolar disorder when compared with controls.

There are few studies assessing neurocognitive functions in Indian subjects with bipolar disorder. These studies have focused on neurocognitive deficits in patients during an episode^8^,^9^ and have not examined neurocognitive deficits in euthymic patients. We therefore aimed to investigate neurocognitive deficits in patients during the euthymic phase of the illness.

## METHODS

### Study group

Thirty patients diagnosed as having bipolar disorder were recruited from the outpatient department of the Schizophrenia Research Foundation. There were 16 males and 14 females. The mean age of this group was 29.60 years (SD 9.68). The mean years of education was 10.97 (SD 3.34). The mean duration of illness was 10.43 years (SD 6.19; range 5–24 years). The average number of episodes was 6.43 (SD 4.35), with a mean of 4.3 (SD 2.93) for manic episodes and 2.33 (SD 2.11) for depressive episodes. Patients were included if they fulfilled the following criteria:

1. ICD-10 criteria for bipolar affective disorder
2. Minimum of 5 years' duration of illness from the first episode
3. Currently in remission as per scores on the Hamilton Rating Scale for Depression (HRSD; score <8) and Young Mania Rating Scale (YMRS; score <11)
4. Age ≤50 years
5. Able to read and write
6. Consent for neuropsychological testing.

Patients with a history of mental retardation, epilepsy, head injury with loss of consciousness, substance abuse, cerebro-vascular disease, neurodegenerative disorders, systemic illness with known cerebral consequences and those not motivated to perform the tests were excluded from the study.

### Control group

The control group comprised 30 subjects (15 males, 15 females), matched with the study group for age, gender and education, with no past, present or family history of psychiatric illness and fulfilling the same exclusion criteria as the study group. The mean age of the control group was 29.37 years (SD 8.10). The mean years of education in the control group was 10.33 (SD 3.29).

### Methods of assessment

All recruited subjects were assessed for psychopathology and neurocognitive functioning using appropriate tools. Psycho-pathology was assessed using HRSD and YMRS. The neuro-psychological test battery included:

- *Digit-symbol test:* To study visuomotor speed and attention
- *Trail-making tests:* Parts A and B from the Halstead–Reitan battery which assesses attention, visuomotor speed (Parts A and B) and executive functioning (Part B)
- *Verbal fluency:* To assess executive function
- *Digit-span test:* To assess immediate verbal memory span (digit forward), auditory attention, short-term retention capacity and manipulation of information in verbal working memory (digit backward)
- *Verbal paired associates:* To measure new learning ability
- *Visual design reproduction:* To assess visual short-term memory and immediate retention
- *Test of logical memory:* To assess short-term learning and memory

Neuropsychological measures of the two groups were compared using the chi-square and Student *t* tests.

## RESULTS

The patients were in remission when recruited into the study. The mean scores on the HRSD and YMRS were 1.4 (range 0–5) and 0.97 (range 0–5), respectively. All patients were on antipsychotics and/or mood stabilizers at the time of assessment.

Statistically significant differences were seen between the study and control groups in the digit-symbol test, trail-making tests A and B, the digit forward test and test of visual memory. The study group performed poorly compared with the control group (Table 1).

**Table 1:** Comparison of performance on various tests between the study and control groups

	Study group (*n*=30)		Control group (*n*=30)		
			
Test	Mean	SD	Mean	SD	*t* test
Digit-symbol	34.10	15.50	45.90	13.98	3.10***
Trail-making (Part A)	62.07	33.40	45.63	17.18	2.40**
Trail-making (Part B)	162.17	68.20	95.83	29.76	4.89****
Verbal fluency	14.37	3.26	13.83	4.63	0.14 (NS)
Digit span—forward	5.23	1.17	5.93	1.37	2.13*
Digit span—backward	3.73	1.18	4.23	1.20	1.61 (NS)
Logical memory	12.57	3.31	13.83	3.00	1.55 (NS)
Paired association learning					
Trial 1 Easy association	3.13	0.92	3.23	0.84	0.44 (NS)
Trial 2 Hard association	0.83	0.78	0.83	1.00	0 (NS)
Trial 3 Hard association	2.47	1.36	2.90	1.17	1.32 (NS)
Visual design reproduction	7.60	3.50	10.77	1.43	4.59****

NS: not significant

* p<0.05;

** p<0.02;

*** p<0.01;

**** p<0.001

## DISCUSSION

This study demonstrates that patients with bipolar disorder in the euthymic phase have neuropsychological impairment. All patients were in remission, as indicated by the HRSD and YMRS. Significant impairments were seen in attention, executive function and memory in the study group compared with a matched control group of normal subjects. This is in keeping with the findings of other studies.^4^,^6^,^7^,^10^

Statistically significant impairments in attention and visuomotor speed as tested by the digit-symbol, trail-making (A and B), digit-span and visual-design reproduction tests were seen in this study. Significant impairment in executive function, tested using the trail-making B test, was also noted. Ferrier *et al*.^4^ reported similar findings, although they used other tests of attention. Impaired attention may have cascading effects on other areas of cognition. Impairment in executive functions indicates frontal lobe damage leading to difficulties in coping with everyday life in terms of goal-setting, selfregulation and decision-making.

The effects of drugs are often a major uncertainty in studies.^11^ All patients in this study were receiving antipsychotics and/or mood stabilizers, which may have had an effect on the cognitive deficits seen in this study. Priority for patient care will restrict the designing of research methodology.^12^

The presence of neuropsychological impairment in patients who have recovered from bipolar disorder is of clinical relevance. It is likely to adversely affect insight, thus increasing the risk for non-adherence. This would lead to manic relapses, thereby causing more cognitive dysfunction. Even minor cognitive deficits may result in substantial social and occupational difficulties. An inability to cope with the demands of work or family could cause stress and thus contribute to relapse. Recognition of this and advice on coping may be of benefit to patients. For functional recovery, a combination of aggressive pharmacotherapy, psychoeducation, cognitive–behavioural therapy and cognitive rehabilitation may be required to manage patients with bipolar disorder.
